# Systematic review of studies comparing infliximab and adalimumab in autoimmune uveitis

**DOI:** 10.1136/bmjophth-2023-001303

**Published:** 2023-06-14

**Authors:** Oliver Mase, Mustafa Qasem, Nicholas Beare

**Affiliations:** 1 School of Medicine, University of Liverpool, Liverpool, UK; 2 St Paul's Eye Unit, Liverpool University Hospitals NHS Foundation Trust, Liverpool, UK; 3 Department of Eye and Vision Science, University of Liverpool, Liverpool, UK

**Keywords:** inflammation, treatment medical

## Abstract

**Objective:**

This study aimed to review effectiveness studies comparing two biological anti-tumour necrosis factor agents, adalimumab (ADA) and infliximab (IFX), in the management of autoimmune uveitis.

**Methods:**

A systematic search was conducted across PubMed, Scopus, Web of Science and Google Scholar from 2014 until February 2022. The search included the following keywords “Adalimumab”, “Infliximab”, “Autoimmune”, “Anterior”, “Intermediate”, “Posterior”, “Panuveitis”, “Refractory” and “Uveitis”. Primary studies comparing both ADA and IFX in a population of autoimmune uveitis patients were considered. Outcomes of interest were measures of response to treatment and incidence of adverse events.

**Results:**

The preliminary literature search generated 7156 references. Six studies fulfilled the eligibility criteria and were included in the final analysis; all were non-randomised, retrospective or observational. The included studies found similar effectiveness and side effect profiles for both ADA and IFX in the management of autoimmune uveitis, however, one did not report effectiveness for each separately, and three were limited to Behcet’s disease.

**Conclusion:**

ADA and IFX seem to display comparable effectiveness and safety profiles. However, the available evidence remains scarce, of low quality and at high risk of bias. A direct comparison between ADA and IFX through large randomised controlled trials is needed to provide more substantial evidence of equivalence or superiority in uveitis.

**WHAT IS ALREADY KNOWN ON THIS TOPIC**

- The use of anti-tumour necrosis factor agents in uveitis is well supported in the ophthalmology literature.

**WHAT THIS STUDY ADDS**

- Recognises the lack of randomised controlled trials that directly compare the performance of adalimumab (ADA) and infliximab (IFX) in the treatment of autoimmune uveitis.
- Discloses that the most effective treatment protocol for ocular inflammatory conditions remains undefined and a research priority.

**HOW THIS STUDY MIGHT AFFECT RESEARCH, PRACTICE OR POLICY**

- A gap in the literature is identified and calls for studies with robust methodologies to directly compare the efficacy and side effect profiles of IFX and ADA for the treatment of autoimmune uveitis.

## Introduction

Autoimmune uveitis refers to a group of disorders that potentially threaten vision through intraocular inflammation and its complications. It is estimated to have an incidence of 17–52 cases per 100 000 person-years,^1^ and account for 10%–15% of preventable visual disability in highly developed countries.^2 3^ Given the immunological nature of this type of uveitis, systemic corticosteroids have long been the mainstay of therapy in acute phases. However, they are often incapable of adequate inflammation control in maintenance doses, and have long-term ocular and systemic adverse effects.^4^ Therefore, there is a large unmet need for effective treatment alternatives in steroid-dependent patients with non-infectious uveitis.

Studies have demonstrated increased levels of tumour necrosis factor alpha (TNF-α) in the serum and aqueous humour of patients suffering with non-infectious autoimmune uveitis.^5^ Persistent, severe or long-term inflammation compelled the introduction of systemic immunomodulatory therapy (IMT) as a means to control the disease, prevent ocular structural damage and allow tapering of corticosteroids. Biologics are used as IMT agents in autoimmune uveitis, and TNF-α inhibitors, in particular, have demonstrated efficacy for ocular inflammation in clinical trials.^3^

Adalimumab (ADA) is a recombinant IgG1 monoclonal antibody specific for TNF-α. It acts by binding directly to TNF-α and blocking its interaction with p55 and p75 cell surface TNF receptors.^6^ Infliximab (IFX) is also a TNF-α inhibitor. It acts by binding to the subunit and precursor of TNF-α, disrupting the activity between TNF-α and its receptor.^7^ Both ADA and IFX act to lyse cells with surface TNF-α through antibody-dependent cell-mediated cytotoxicity and/or complement-dependant cytotoxicity. The drugs aim to reduce intraocular TNF-α, and hence inflammation in the eye. ADA is approved for use in autoimmune uveitis by the Food and Drug Association and European Medicines Agency. Although approved for use in other inflammatory conditions, IFX remains unlicensed for use in non-infectious uveitis by either body. Nonetheless, multiple international experts recommend IFX in the management of sight-threatening Behcet’s disease (BD).^8^

The use of anti-TNF-α agents in the treatment of inflammatory, non-infectious uveitis is supported by a wide international literature.^3 9^ In the case of ADA, this includes randomised controlled trials against placebo.^3^ However, comparative data remain lacking. This review aims to assess the current body of evidence that compares the efficacy and safety of IFX and ADA in the treatment of autoimmune uveitis.

## Methodology

### Literature search

This review of literature aimed to comply with the Preferred Reporting in Systematic Reviews and Meta-Analysis guidance.^10^ We searched the published literature on the following databases from 2014 to February 2022: PubMed/Medline, Scopus, Web of Science and Google Scholar. The search was aimed at publications comparing the efficacy and safety profiles of both ADA and IFX in patients with uveitis. The following terms were used in isolation and/or combination: “Adalimumab”, “Infliximab”, “Autoimmune”, “Anterior”, “Intermediate”, “Posterior”, “Pan-uveitis”, “Refractory” and “Uveitis”. The term “Treat*” was added to the search terms when using Google Scholar to account for the relative low precision of the search engine.

### Inclusion and exclusion criteria

The eligibility of the search results was evaluated by one reviewer (OM) against the predefined set of inclusion/exclusion criteria. The eligibility criteria adopted for this review conformed to the PICOS (population, intervention, control, outcomes and study design) model. Studies included described administration protocols of both ADA and IFX on a sample size larger than 40 adult patients with non-infectious, inflammatory uveitis. Outcomes of interest were rates of adverse events and any measure of treatment effectiveness; including improvements in clinical measures of inflammation, best corrected visual acuity (BCVA), macular thickness and corticosteroid sparing effects. Randomised controlled trials, cohort and observational studies comprised the intended literature for this review. Review articles and studies assessing either drug independently without drawing a direct comparison were excluded. Articles included were limited to those published in the English language.

### Data extraction

One author (OM) reviewed and retrieved the studies. Articles were primarily screened through their title and abstract. A full-text evaluation was then performed to identify the most relevant studies to the objective of this review. Extracted data included: study identifier, study design, number of participants, intervention applied and outcomes measured.

### Quality assessment

The primary outcomes reviewed were assessed against the Grading quality of evidence and strength of recommendations (GRADE) system.^11^ Although the quality of evidence represents a continuum, the GRADE approach identifies four grades for the quality of any body of evidence: high, moderate, low and very low.

## Results

### Study selection

**Figure 1:**
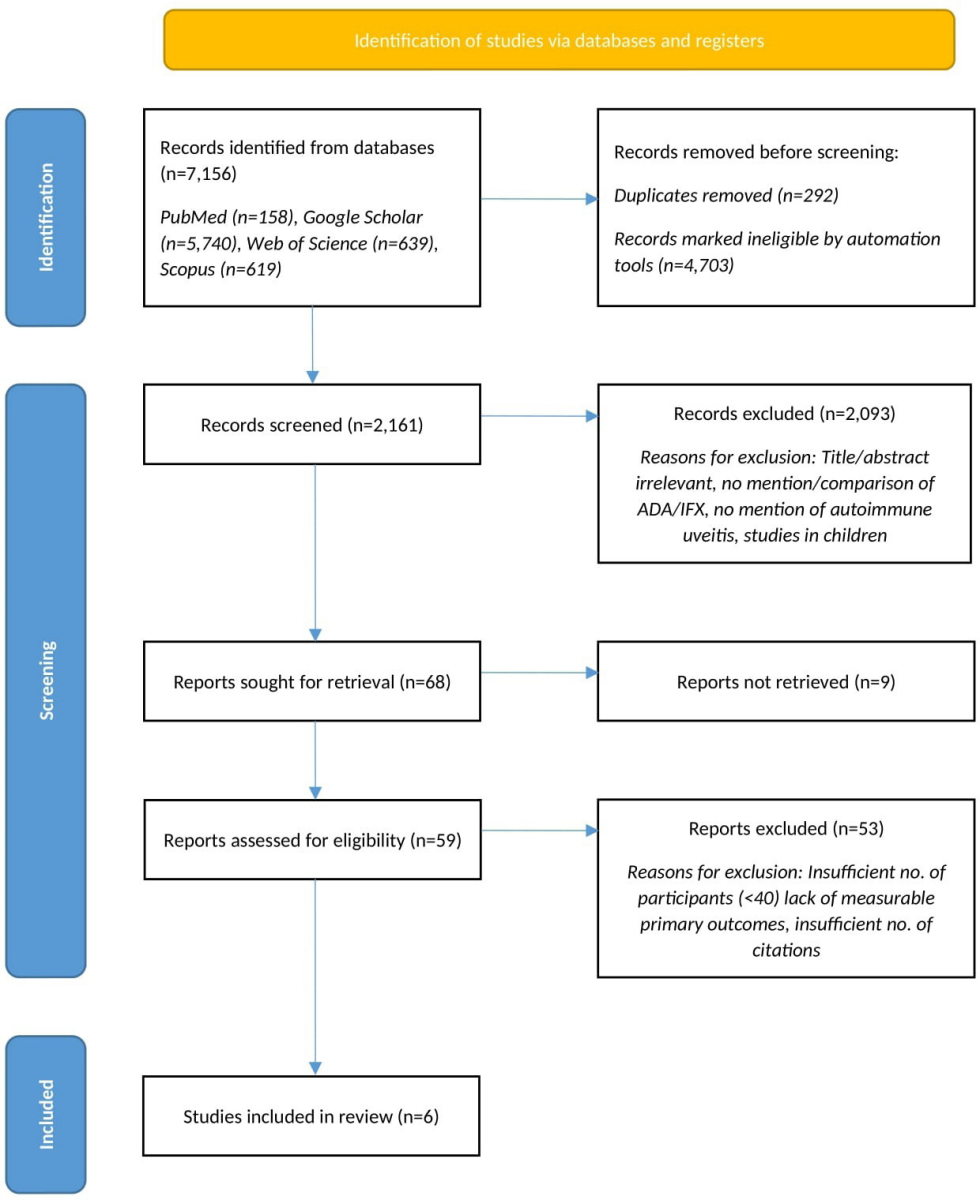
PRISMA 2020 flowchart. ADA, adalimumab; IFX, infliximab; PRISMA, Preferred Reporting in Systematic Reviews and Meta-Analysis.

An extensive literature search yielded 7156 references (see table 1). Application of the inclusion criteria via automation tools reduced the number to 1908 potentially relevant, non-duplicate studies. Automation tools were used to limit the papers found across all databases to those published between 2014 and 2022 and review articles. The search on Scopus also included research articles. Web of Science and PubMed additionally limited the search to papers published in English, and PubMed only included Human studies. The papers were subsequently screened through a review of title and abstract. Sixty-eight studies were retrieved for comprehensive full-text analysis. Following evaluation, six articles were found to be closely related to the objective of this review and were included in the final analysis (see figure 1).

**Table 1:** Search terms used and results

Search terms	Number of search results for each database			
	Web of Science	Scopus	PubMed	Google Scholar
“Adalimumab” AND “Infliximab”	6092	19 144	5075	73 500
“Adalimumab” AND “Infliximab” AND “autoimmune uveitis”	6	34	4	691
“Adalimumab” AND “Infliximab” AND “anterior uveitis”	76	115	51	3880
“Adalimumab” AND “Infliximab” AND “intermediate” AND “uveitis”	28	100	15	2560
“Adalimumab” AND “Infliximab” AND “posterior uveitis”	48	43	12	1990
“Adalimumab” AND “Infliximab” AND “panuveitis”	36	66	26	2260
“Adalimumab” AND “Infliximab” AND “refractory uveitis”	112	61	31	1750
“Adalimumab” AND “Infliximab” AND (“autoimmune” OR “anterior” OR “intermediate” OR “posterior” OR “panuveitis” OR “refractory” AND “uveitis”)	**639**	**619**	**158**	10 600
“Adalimumab” AND “Infliximab” AND “treat*” AND (“autoimmune” OR “anterior” OR “intermediate” OR “posterior” OR “panuveitis” OR “refractory” AND “uveitis”)	n/a	n/a	n/a	**5740**

### Study characteristics

The study designs used to investigate ADA and IFX in autoimmune uveitis included two open-label studies, three retrospective studies and one prospective cohort study (see table 2). Evidence was collated from 735 patients from a wide background of immune-mediated uveitic subtypes.

**Table 2:** Individual study characteristics for studies comparing adalimumab to infliximab in autoimmune uveitis

Author, year	Location	Single/multicentre	No. of participants	Type of study	Results
Vallet *et al* (2016)^12^	France	Multicentre	160 participants 98 received IFX 62 received ADA	Retrospective study in refractory uveitis	Response rate (complete or partial) seen in 97% IFX group and 95% ADA group IFX and ADA had significant corticosteroid sparing effects (50% reduction at 6 months in both groups) 16% of IFX group and 6% of ADA group experienced serious side effects IFX and ADA have equivalent effectiveness and side effect profile ADA superior for long-term remission
Vallet *et al* (2015)^14^	France	Multicentre (21 internal medicine and rheumatology referral centres)	124 participants 77 received IFX 37 received ADA	Retrospective study in Behcet’s disease	Improvement of uveitis seen in 96.4% of IFX group and 95.7% of ADA group Relapse-free survival similar for both groups Both drugs had corticosteroid sparing effects No significant difference between the effectiveness or safety of ADA and IFX
Atienza-Mateo *et al* (2019)^17^	Spain	Multicentre (52 uveitis referral units in Spanish hospitals)	177 participants 103 received IFX 74 received ADA	Observational, open-label study in Behcet’s disease	After 12 months—improvements in all ocular parameters in both groups ADA had significantly better outcomes in: Improvement of vitritis (93% vs 79%) BCVA (mean±SD 0.82 vs 0.67±0.34). Drug retention rate (71% vs 44%) Similar improvement between ADA and IFX groups in: Improving anterior chamber inflammation Macular thickness Improvement of retinal vasculitis IFX and ADA are effective (although ADA may be associated with better outcomes)
Calvo-Rio *et al* (2014)^15^	Spain	Multicentre (38 uveitis referral units in Spanish hospitals)	124 participants 77 received IFX 47 received ADA	Observational, open-label study in Behcet’s disease	At 12 months—rapid and maintained improvement in all ocular parameters in the whole cohort: Intraocular inflammation Macular thickness and visual acuity Sparing effect of corticosteroids Immunosuppression load
Fabiani *et al* (2018)^18^	Italy	Multicentre	107 participants 41 received IFX 66 received ADA	Retrospective study	Decrease of uveitis flare frequency in 12 months 84.2% (ADA) and 66.7% (IFX) IFX more effective at reducing uveitic macular oedema and macular thickness IFX had significantly better corticosteroid sparing effects ADA and IFX have similar effectiveness in controlling uveitis relapses
Sharma *et al* (2019)^16^	UK	Single centre	43 participants 34 received IFX nine received ADA	Prospective cohort study	67% of ADA group achieved sustained remission 100% later experienced a relapse (median 2 years from start of treatment) 97% of IFX group achieved sustained remission 54% later experienced a relapse (median 3.4 years from start of treatment)

ADA, adalimumab; BCVA, best corrected visual acuity; IFX, infliximab.

Vallet *et al*^12^ retrospectively compared the effectiveness and safety of ADA and IFX in patients with non-infectious refractory uveitis across multiple centres. Of the 160 patients identified, 98 were treated with IFX and 62 with ADA. Response to treatment was defined as an improvement of at least 50% in the level of inflammation—evaluated according to the SUN Working Group criteria,^13^ and/or significant regression of retinal vasculitis and macular oedema, and a reduction of at least a 50% in the initial corticosteroid dosage at 6 months of therapy. The response was achieved in 97% of the patients treated with IFX and 95% of those treated with ADA. Adverse events were reported in 28% of the patients, with 13% being severe enough to cause treatment discontinuation. The most frequent side effects encountered were infection, hypersensitivity reactions and autoimmune disease. This study reports on the side effects of the entire cohort and does not draw a direct comparison between the two drugs in that regard. However, it concludes that the two agents are of equivalent effectiveness in achieving a complete response and with no significant difference in the cumulative incidence of serious side effects when used as part of the management of refractory inflammatory uveitis.

Vallet *et al*^14^ assessed the long-term effectiveness and safety of IFX and ADA in patients with severe and/or refractory uveitis in BD, through a multicentre retrospective observational study. The study sample comprised 124 participants, 77 of which received infliximab and 37 received ADA. The study found that 96.4% and 95.7% of its sample responded to IFX and ADA, respectively, with response to treatment defined according to the same criteria as Vallet *et al*.^12^ The most common side effect experienced across the patient population was infections, occurring in 14.3% of the patients receiving IFX, and in 13.5% of those receiving ADA. Although hypersensitivity reactions occurred more frequently with ADA, it did not reach statistical significance. Hence, the effectiveness and tolerance profiles of ADA and IFX were concluded to be similar in the treatment of severe and/or refractory uveitis in patients with BD.

Calvo-Rio *et al*^15^ conducted an open-label, interventional case-series to assess the clinical response of refractory uveitis in patients with BD to anti-TNF therapy. Of the 124 patients selected, 77 received IFX and 47 received ADA. The study reported a significant, rapid and maintained improvement in intraocular inflammation, macular thickness and BCVA. The biological therapy was well tolerated by most patients, with no discontinuations of therapy in any instance. It was concluded that IFX and ADA are effective and relatively safe in refractory uveitis due to BD. However, the study made no direct comparison between the individual drugs, and the outcomes were measured across the entire cohort.

Sharma *et al*^16^ studied 43 patients with non-infectious uveitis or scleritis through a longitudinal cohort design. Of the study sample, 34 were treated with IFX and 9 with ADA. The study resulted in a rate of sustained remission (over two visits) of 97% in the IFX group, with relapse occurring at a rate of 53% at a median of 3.4 years. Conversely, remission was sustained in 67% of the ADA group, all of whom later experienced a relapse at a median of 2.0 years. Both drugs had a significant corticosteroid sparing effect, with 39% of the participants able to withdraw steroid use completely. The rate of adverse effects was similar between both groups with 54% of the participants experiencing at least one adverse effect.

A multicentre, open-label, observational study compared the effectiveness, safety and retention rates of IFX and ADA in the treatment of patients with refractory uveitis due to BD. Atienza-Mateo *et al*^17^ enrolled 177 patients, 103 of whom received IFX, and 74 received ADA. At 12 months, improvement was noted in all ocular parameters for both groups. Nevertheless, the ADA group displayed significant improvement in vitritis grade (in 93% of the ADA group vs in 79% of the IFX group), and BCVA (mean±SD 0.82 in the ADA group compared with 0.67±0.34 in the IFX group). Toxicity severe enough to discontinue treatment occurred in 7% of the ADA group, compared with 5% in the IFX group. However, patients receiving ADA demonstrated a higher drug retention rate of 71% versus 44% in the IFX group. The study thus concluded that ADA displays significant superiority with regard to improvement in vitritis, BCVA and drug retention rate.

Finally, a retrospective study by Fabiani *et al*^18^ compared the effectiveness of the two agents in the management of 107 patients with non-infectious intermediate uveitis, posterior uveitis or panuveitis. Sixty-six patients received ADA and 41 received IFX, with a primary aim to assess the overall frequency of uveitis relapses over 12 months of treatment. IFX and ADA displayed similar effectiveness in suppressing ocular relapses with an overall decrease in uveitis frequency of 67% and 84%, respectively. Moreover, both drugs displayed similar ability in controlling retinal vasculitis and preserving visual function. Conversely, IFX demonstrated a more marked corticosteroid sparing effect, and was able to significantly reduce rates of uveitic macular oedema as compared with ADA.

### Confidence in cumulative evidence

According to the GRADE system, the available body of evidence is of very low quality.^11^ The unrandomised, observational or retrospective study designs lack an inherent ability to control for confounding factors which is the main limiting factor of evidence quality. In addition, the indirectness of evidence due to heterogeneity in study populations and outcome measures are limiting. As such, there is low confidence in the hypothesis that ADA and IFX are comparable in efficacy and safety profiles in the treatment of autoimmune uveitis.

## Discussion

It is apparent through this systematic review of the literature that ADA and IFX have not been directly compared in a randomised trial as treatments for autoimmune uveitis. Randomised controlled trials have demonstrated ADA has efficacy in uveitis compared with placebo,^3 19 20^ and we have found some evidence that ADA and IFX have similar effectiveness and safety but that evidence is of low quality and subject to significant biases. Each of the six papers identified concludes that both anti-TNF-α agents have some degree of impact on decreasing the inflammatory activity and improving the respective ocular parameters measured. However one paper did not report outcomes for each medication separately and three of the studies are in BD, leaving just two observational studies which compared ADA to IFX in autoimmune uveitis.

While the majority of the references reviewed conclude no significant difference in effectiveness, Atienza-Mateo *et al*^17^ found superior outcomes in some parameters at 12 months of therapy in patients receiving ADA. In contrast, Vallet *et al*^14^ report greater corticosteroid sparing effects and reduction in macular oedema with IFX use. This corresponds with the findings of Levy-Clarke *et al*^21^ who also suggest that IFX shows an increased ability to resolve uveitic macular oedema compared with ADA.

Atienza-Mateo *et al*^17^ revealed IFX to be likely associated with a higher incidence of adverse events as compared with ADA. This was represented by a significantly higher drug retention rate of 71.62% in the ADA group versus 44.12% in the IFX group. This is thought to be due to the murine component of IFX which can cause infusion reactions, and stimulate anti-IFX antibodies.^7 22^ Nonetheless, the study acknowledges that their rates of discontinuation may have also been influenced by patient’s preference to a different route of administration (subcutaneous for ADA, intravenous for IFX), achieving remission or loss of effectiveness of treatment.

The six papers reviewed administered 3–5 mg/kg of IFX every 4–8 weeks after the initial loading dose and 40 mg of ADA every 1–2 weeks regardless of body weight. A study by Sukumaran *et al*^23^ showed that increasing the dose of IFX to ≥10 mg/kg improved disease control for an additional 35% of patients who did not respond to the initial treatment. In addition, Vallet *et al*^14^ found that switching anti-TNF-α agents due to lack of effectiveness or adverse effects provided a further complete or partial response in 67% of 37 patients at 6 months, although this methodology is fraught with biases. These findings concur with those by Olivieri *et al*^24^ following a study to assess the efficacy of ADA after unsuccessful treatment with IFX, with 71% of the participants demonstrating response to treatment, with no side effects observed.

The limitations of this review are predominantly secondary to the poor design of the articles within its scope. The retrospective design that predominates the literature on the comparative efficacy of ADA and IFX in autoimmune uveitis, introduces a significant risk of bias. Significant heterogeneity was observed among the study variables. The patient population lacks consistency across the studies identified, and there is an evident absence of standardisation in the assessment of treatment efficacy of uveitis, with studies adopting diverse outcome measures. This further limited the ability of this review to compare and draw conclusions from the available body of evidence. Only one author identified studies for inclusion, but with broad inclusion applied liberally, we are confident to have included all significant studies comparing these two medications.

## Conclusion

The current body of literature suggests that both ADA and IFX have equivalence in treating autoimmune uveitis, with similar safety profiles. However, available evidence remains severely limited by risk of bias and inadequate study designs. It is clear that a randomised controlled trial of ADA and IFX is needed to ascertain if they are equivalent, or one is superior in autoimmune uveitis. Establishing the most effective treatment for ocular inflammatory disease is the first research priority of the James Lind Alliance for inflammatory eye disease.^25^ Now that IFX is available as a subcutaneous injection, a direct and masked comparison can be made between it and ADA. More research is also needed to determine the optimal dosing of both anti-TNF agents, and whether dose escalation can be beneficial in order to achieve a higher overall response rate. Furthermore, the response rates in different uveitis entities would benefit treatment decision-making in patients affected by uveitis.

## Data Availability

Data are available in a public, open access repository.
